# Dasatinib self-assembled nanoparticles decorated with hyaluronic acid for targeted treatment of tumors to overcome multidrug resistance

**DOI:** 10.1080/10717544.2021.1905751

**Published:** 2021-04-01

**Authors:** Yawen Zhang, Xiangle Zeng, Hairong Wang, Ranran Fan, Yike Hu, Xuejie Hu, Jianchun Li

**Affiliations:** School of Pharmacy, Bengbu Medical College, Bengbu, China

**Keywords:** pH response, multidrug resistance, dasatinib, hyaluronic acid, micelle

## Abstract

Multidrug resistance (MDR) and lack of targeting specificity are the main reasons why traditional drug therapies fail and produce toxic side effects in cancer chemotherapy. In order to increase targeting specificity and maximize therapeutic efficacy, new intelligent drug delivery systems are needed. In this study, we prepared the hyaluronic acid (HA) conjugated dasatinib (DAS) and D-α-tocopherol acid polyethylene glycolsuccinate (TPGS) copolymer nanoparticles (THD-NPs). The water solubility of the hydrophobic drug DAS was improved by chemically linking with HA. HA can bind to the over-expressed CD44 protein of tumor cells to increase targeting specificity, TPGS can inhibit the activity of P-glycoprotein (P-gp), and increase the intracellular accumulation of drugs. The prepared drug-loaded nanoparticle has a particle size of 82.23 ± 1.07 nm with good *in vitro* stability. Our *in vitro* studies showed that THD-NPs can be released more rapidly in a weakly acidic environment (pH = 5.5) than in a normal physiological environment (pH = 7.4), which can realize the selective release of nanoparticles in tumor cells. Compared to free drugs, THD-NPs showed more efficient cellular uptake, effectively increased the cytotoxic effect of DAS on nasopharyngeal carcinoma HNE1 cells drug resistance HNE1/DDP cells and increased the accumulation of drugs in HNE1/DDP cells, which may be due to the inhibitory effect of TPGS on the efflux function of P-gp. *In vivo* experiments showed that THD-NPs can effectively inhibit tumor growth without obvious side effects. In conclusion, the targeted and pH-sensitive nanosystem, we designed has great potential to overcome drug resistance and increase therapeutic effects in cancer treatment.

## Introduction

Dasatinib (DAS) is a second-generation tyrosine kinase inhibitor. As a multitarget small molecule drug, it can target a variety of tyrosine kinases related to tumor cell growth. The most sensitive targets include BCR-ABL, SRC family, receptor tyrosine kinases (c-KIT, PDGFR, DDR1), and TEC family kinases, etc (Montero et al., 2011; Lindauer & Hochhaus, 2018). As the first-line drug for chronic myelogenous leukemia (Levêque et al., 2020), DAS is 300 times stronger than imatinib in inhibiting BCR/ABL activity (Lindauer & Hochhaus, 2010). Recent studies have found that the drug can inhibit cancer cell replication, migration, invasion, and trigger tumor cell apoptosis (Lindauer & Hochhaus, 2018). It has shown obvious therapeutic effects in a variety of solid tumors, and research on breast cancer has entered Clinical Trials. However, DAS has poor water solubility, absorbs easily affected by the pH, and its terminal half-life is only 3–4 h (Horinkova et al., 2019). At the same time, there are a series of side effects (Niza et al., 2019). Therefore, it is necessary to design a suitable drug carrier, in order to achieve safe and efficient delivery of DAS, improve its efficacy and reduce adverse reactions.

In addition to the development of new targets for modern antitumor drug therapy, the development of new drug delivery systems is also particularly important. With the development of nanometer medicine, Nano Drug Delivery System (NDDS) has shown superior performance (Ren et al., 2021; Tian et al., 2021; Zhang et al., 2021; Zhou et al., 2021). Through different modifications to the NDDS carrier, can make its active targeting and environmental responsiveness (Zhang et al., 2021; Zhou et al., 2021). As a type I transmembrane glycoprotein, CD44 protein is widely expressed in endothelial cells, mesenchymal cells, and mesoderm-derived cells. It can promote dynamic interactions inside and outside cells, cell movement, and transfer. Because of its abnormal expression in tumor cells, it is often used as a tumor treatment target to prepare active targeting agents (Tian et al., 2017). As a natural polysaccharide, hyaluronic acid (HA) is one of the main CD44 ligands. The specific binding of HA to CD44 can make HA-based nanodrug delivery system localize and concentrate on tumor sites. At the same time, HA has excellent biocompatibility, hydrophilicity, low immunogenicity, chemical modifiability, and biodegradability, making it an ideal material for targeted drug delivery systems (Williams et al., 2013; Karousou et al., 2017; Tirella et al., 2019; Yao et al., 2019; Li et al., 2021).

Multidrug resistance (MDR) is one of the main obstacles to effective chemotherapy of tumor. Mechanisms of MDR production include increased efflux of drugs to reduce intracellular accumulation of drugs, promoting antiapoptotic mechanisms, and repairing DNA damage (Almalik et al., 2013; Koshkin et al., 2013; Singh et al., 2017; Trujillo-Nolasco et al., 2019). Among them, P-glycoprotein (P-gp), as a membrane transporter of the ATP binding cassette family, can pump out substrate drugs through an ATP-dependent mechanism, thereby reducing intracellular drug accumulation, which is one of the main causes of MDR (Chen et al., 2016; Gupta et al., 2017; Li et al., 2017). A variety of P-gp inhibitors such as Pluronic, Verapamil, and TPGS have been discovered (Gottesman et al., 2002; Negi et al., 2019; Xu et al., 2020). Among them, D-α-tocopherol acid polyethylene glycolsuccinate (TPGS), an amphiphilic structure, can self-assemble to form nanoparticles and improve the permeability to cancer cells, making it an ideal nanocarrier (Wang et al., 2016; Popova et al., 2019; Tao et al., 2020).

**Figure 1.**
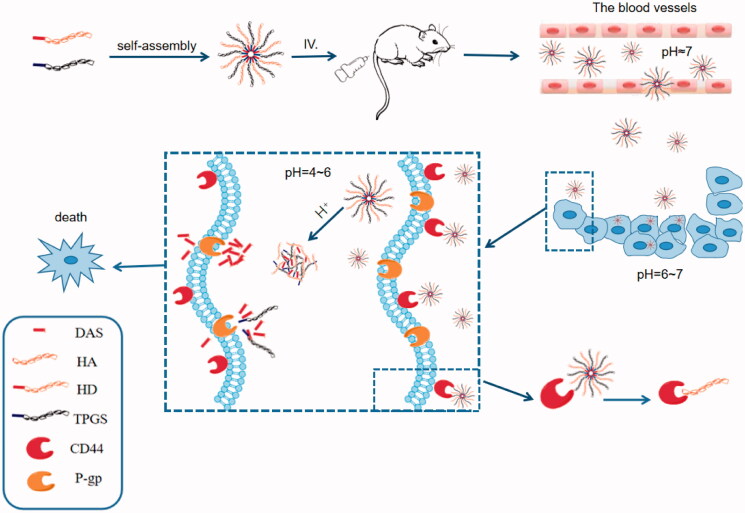
The construction, *in vivo* release and mechanism of THD-NPs. THD-NPs enter the systemic blood circulation through intraperitoneal injection. The CD44 receptor overexpressed on the surface of tumor cells specifically binds to the hyaluronic acid of THD-NPs, and the drug is transported into tumor cells through receptor-mediated endocytosis. THD-NPs disintegrate in response to low pH in tumor cells and release drugs. TPGS inhibits P-gp function to reduce drug efflux, increase intracellular accumulation of drugs, cause cancer cells to die and reduce toxicity to normal cells.

In this study, we designed to link the hydroxyl group of DAS and the carboxyl group of HA through ester construction to obtain an amphiphilic complex with DAS as the hydrophobic segment and HA as the hydrophilic segment. HA acts as a ligand for CD44 protein, which can target tumor cells overexpressing CD44 receptor, thus enabling targeted drug delivery (Gao et al., 2019). At the same time, the addition of TPGS to the carrier increases the stability of the nanoparticles, inhibits the P-gp activity, and increases the intracellular accumulation of the drug. Due to the abnormal metabolism of tumor cells, the pH of tumor tissues is lower than that of normal tissues (Wan et al., 2020), and the ester in DAS-HA complex can be broken in response to the weak acidic microenvironment in tumor cells, thereby releasing the drug. Briefly, this experiment proposes to design a pH-sensitive targeted nanoparticle to achieve the targeted release of DAS, and increase its intracellular accumulation, to provide a safe and efficient agent for DAS (Figure 1).

## Materials and methods

### Chemicals

DAS and TPGS were from Macklin Biochemical Technology Co. Ltd. (Shanghai, China), and HA (MWCO: 8814 Da) was provided by Bloomage Biotechnology Corporation Limited (Shandong, China). EDC and DMAP were purchased from Aladdin Co. Ltd. (Shanghai, China). HA-DAS complex was prepared by the research group, phosphate-buffered saline (PBS) obtained from Saiguo Biotechnology Co. Ltd. (Hangzhou, China), Dialysis bags with a molecular weight cutoff of 3.5KDa (Viskase Companies, Inc., Darien, IL) were used. RPMI 1640 medium was acquired from Thermo Fisher Biochemical Products (Beijing, China). Fetal bovine serum was supplied by Tian Hang Biotechnology Co. Ltd. (Hangzhou, China). HNE1/DDP cell line and HNE1 cell line was purchased from Beijing Beina Chuanglian Biotechnology Research Institute, and was cultured and stored in the Laboratory of Biochemistry and Pharmacology, Bengbu Medical College.

### Cell culture and animals

HNE1 cells were cultured in RPMI 1640 medium containing 1% penicillin and streptomycin, 10% fetal bovine serum at 37 °C and 5% CO_2_ in humidified incubator. HNE1/DDP cells were cultured in RPMI 1640 medium containing 1% penicillin and streptomycin, 1 μg/mL cisplatin, 10% fetal bovine serum at 37 °C and 5% CO_2_ in humidified incubator. BALB/C nude mice was obtained from Cavens lab animal company (Jiangsu, China), and raised in a laboratory without a specific pathogen barrier.

All animal procedures comply with animal ethics requirements. The animal experimental procedure was approved by the Animal Care Committee of Bengbu Medical College (License No.: 2018033) on 19 January 2018 and conformed to the Animal Ethical Standards and Use Committee at Bengbu Medical College.

### Preparation and characterization of self-assembly nanoparticle

The connection of DAS and hyaluronic acid (HA-DAS) referred to the previous preparation method of the research group. In short, in the presence of the catalysts EDC and DMAP, the carboxyl group of HA is linked to the hydroxyl group of DAS.

**Figure 2.**
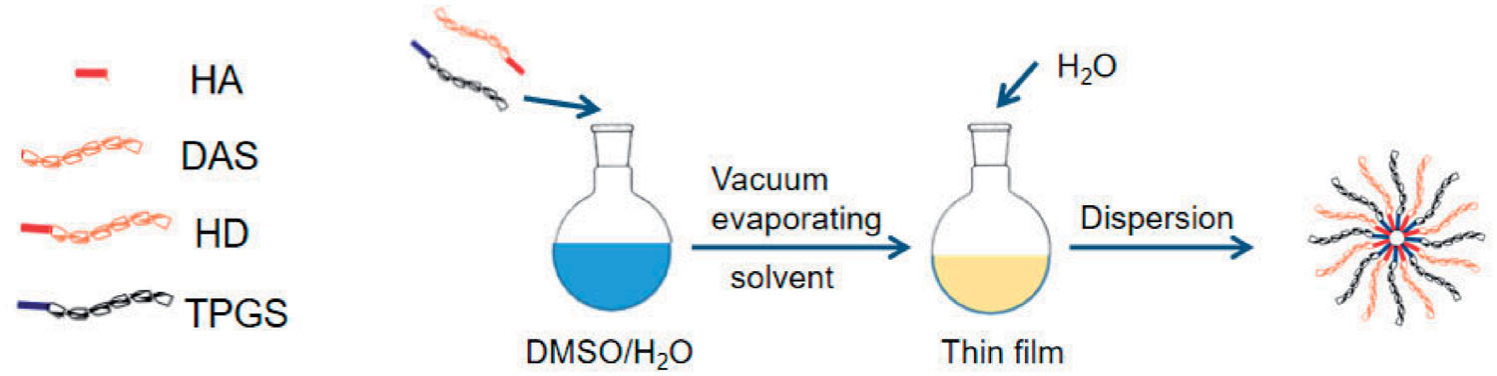
The preparation of THD-NPs.

In order to synthesize HA-DAS(HD)/TPGS mixed micella (THD-NPs), the thin films of polymer and TPGS were first established, and then the thin films were hydrated (Cheng et al., 2018). During the preparation, a total of 20 mg HA-DAS and TPGS were taken and dissolved with water and methanol. After ultrasonication in an ice bath for 30 min, the solvent was removed using a rotary evaporator at 35 °C. The micelle solution was obtained by dispersing the film with water and filtering it through a 0.22 μm filtration membrane (Figure 2).

Zeta Sizer Nano series Nano-ZS (Malvern Instruments Ltd., Malvern, UK) was used to evaluate the THD-NPs size distribution. Characterization of THD-NPs particle morphology was performed using transmission electron microscopy (TEM).

### Evaluation of drug loading

The ester structure was broken by a strong base hydrolysis method to determine the DAS content. The mixture of 1 mL micelle solution and 100 μL NaOH solution (1 mol/L) was fully reacted for 30 min, then 100 μL of HCl solution (1 mol/L) was added to neutralize the solution. Finally, 1 mL methanol was added for ultrasonic demulsification at 200 W power for 5 min. From this, the DAS concentration was determined by HPLC. The drug loading was calculated based on Equation (1)

(1) $$\text{DL}\%=W_{1}/W*100\%$$

W_1_：mass of drug in micelle

W：mass of the mixed micelle

### Characterization of the stability of THD-NPs

The THD-NPs were incubated with 10% fetal bovine serum (FBS)/PBS (pH 7.4) at room temperature and sampled at set time points, then the size distribution was evaluated by Zeta Sizer Nano Series Nano-ZS to investigate the stability of THD-NPs *in vitro*.

### Evaluation of *in vitro* drug release kinetics

In order to evaluate the feasibility of THD-NPs release *in vivo*, PBS of different pH were used to simulate the normal physiological environment (pH = 7.4) and tumor microenvironment (pH = 5.5), and the drug release profile of THD-NPs was determined by dialysis (Priya et al., 2021). Briefly, THD-NPs (3 mL) were added to dialysis bags (MWCO: 3.5 KDa) and placed in PBS with different pH values. The PBS (1 mL) was withdrawn at different time interval and replenish the same Volume of PBS. The content of DAS in each dialysate was measured by HPLC.

### *In vitro* cellular uptake of nanoparticles and DAS accumulation

HNE1/DDP cells (1 × 10^5^ cells/well) were inoculated into a six-well plate and cultured overnight. After incubation with coumarin-6, TPGS loaded with coumarin-6 (TC-NPs) and TPGS/HA-DAS loaded with coumarin-6 (THDC-NPs) at 37 °C and 5% CO_2_ for 2 h, the cells were washed with PBS solution, fixed with 4% paraformaldehyde for 15 min, and stained with DAPI for 10 min. Observe with fluorescence microscope.

HNE1/DDP cells (5 × 10^3^ cells/well) were seeded in 24-well plates and incubated with THD-NPs and HD-NPs at 37 °C for 1, 2, and 4 h, respectively, at a concentration of 50 μM (as DAS content). The cells were centrifuged at 4 °C, and 200 μL of cell lysate was added to the lower precipitate and incubated in an ice bath for 30 min. The supernatant was removed by centrifugation again at 14,000 rmp for 10 min at 4 °C, and the supernatant was used for protein quantification. Finally, the supernatant (100 μL) was mixed with methanol (300 μL) by ultrasonic extraction, centrifuged at 5000 rpm for 10 min, and the supernatant was collected for DAS concentration determination by HPLC. The drug concentration per unit of protein was determined (Bhattacharya et al., 2020).

### Vitro verification of anticancer activity of nanoparticles

The MTT method was used to examine the *in vitro* cytotoxicity of THD-NPs. HNE1 and HNE1/DDP cells in the logarithmic growth phase were seeded in a 96-well plate at a density of 5 × 10^3^ cells/well for 24 h, then the cell culture medium was replaced with 100 μL medicated medium containing different concentrations of DAS and THD-NPs. After 24 and 48 h of incubation, added 20 μL of MTT (5 mg/mL in PBS) to each well, removed the solution after incubating for 4 h, and added 200 μL of DMSO, measured the absorbance at 490 nm after incubated at 37 °C for 30 min.

### Antitumor effects of nanoparticles *in vivo*

To evaluate the antitumor effect of THD-NPs *in vivo*, we established a HNE1 solid tumor model, for that HNE1 cells (5 × 10^6^) were subcutaneously injected into the right upper limb of BALB/C nude mice. The tumor-bearing mice were randomly divided into three groups (control group, DAS group, THD-NPS group, *n* = 3/group). When the tumor volume grew to about 100 mm^3^, each group was intraperitoneally injected with the corresponding drug preparations every 2 days for 2 weeks. The body weight and tumor volume of the mice were recorded during the administration. After 2 weeks of continuous administration, the tumor tissues were extracted and weighed. To evaluate the biosafety of THD-NPS, organs such as heart, liver, lung, spleen, and kidney were extracted for HE staining.

### Statistical analysis

All experimental values were expressed as mean ± SD and all experiments were performed independently and repeated at least three times. Statistical comparisons of data sets were evaluated using the one-way ANOVA analysis. *p* < .05 was considered statistically significant.

## Results and discussion

### Characterization

**Figure 3.**
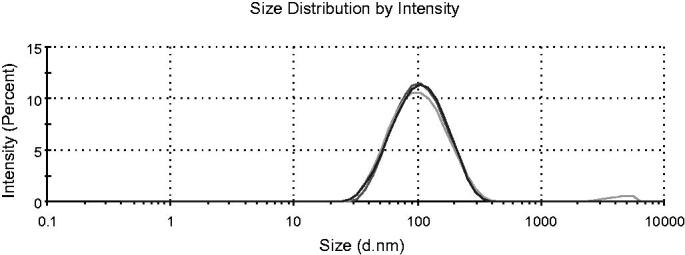
Size of THD-NPs. Dydrodynamic size distribution of THD-NPs, different colors represent the results of three tests (*n* = 3).

**Figure 4.**
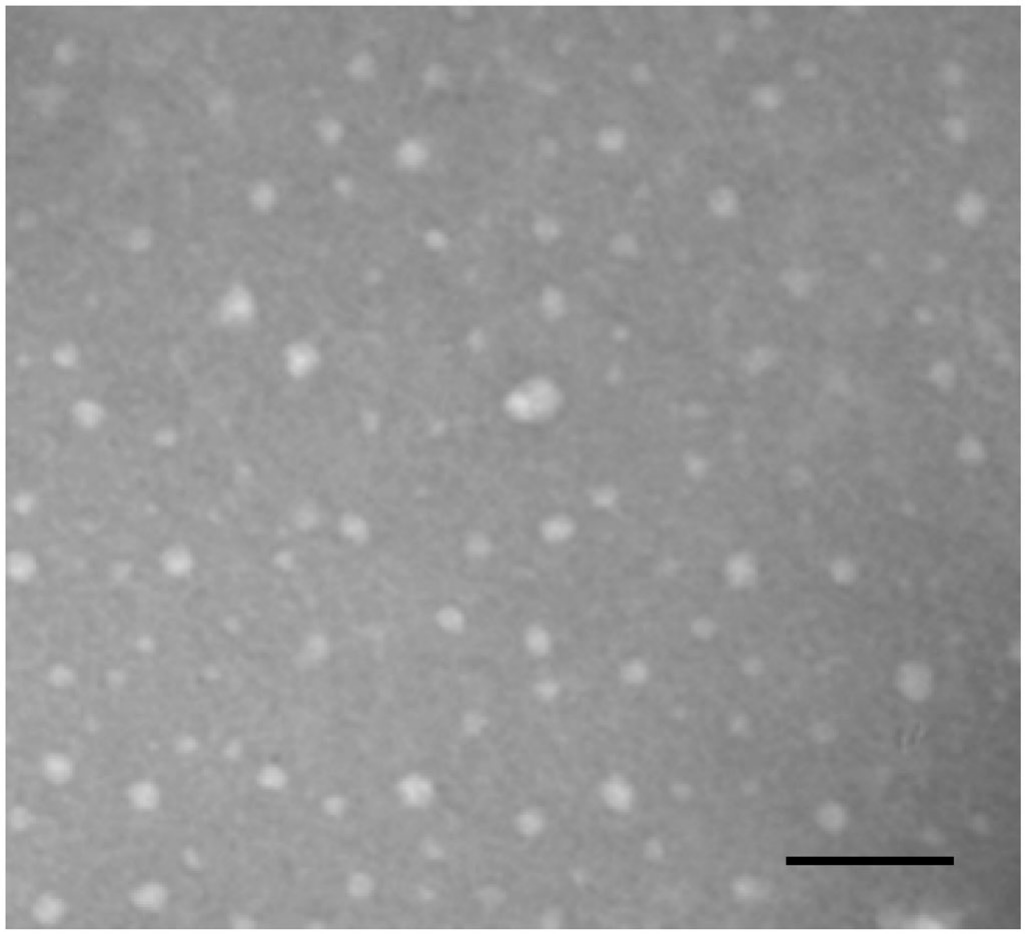
TEM characterization of THD-NPs. Scale bar = 1 μm.

Firstly, we characterized the physicochemical parameters of the THD-NPs. The proportion of HA-DAS and TPGS affects the particle size of the micelle. As the proportion of TPGS increases, the micelle particle size decreases gradually, while the drug load of DAS also decreases correspondingly. After comprehensive consideration, HA-DAS: TPGS is finally selected as 6:1. The results showed that the hydromechanical diameter of THD-NPs was 82.23 ± 1.07 nm and PDI was 0.33 ± 0.004 (Figure 3; Table 1). TEM maps show that THD-NPs are uniformly spherical (Figure 4).

**Table 1.** Average size and polydispersity index (PDI) of THD-NPs.

Sample	Size (nm)	PDI
THD-NPs	82.23 ± 1.07	0.33 ± 0.004

The data represented as the mean ± SD (*n* = 3).

After determining the optimal prescription, free DAS was released by destroying the ester compound, and the drug loading was determined by HPLC. The results showed that the drug loading was 2.1%.

**Figure 5.**
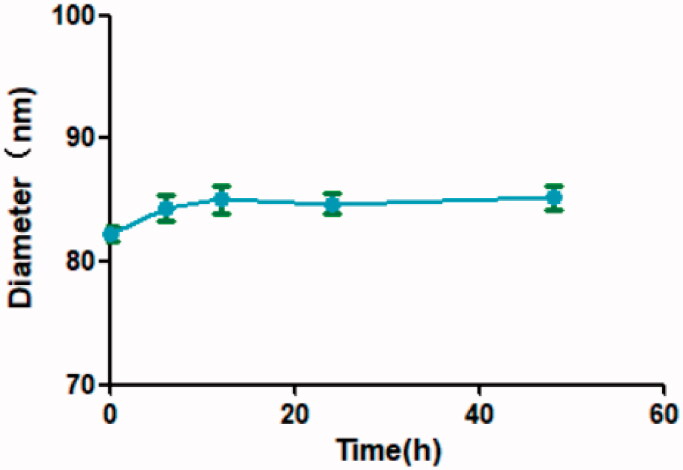
Graphical representation of hydrodynamic diameter. THD-NPs were incubated with an equal volume of 20% FBS/PBS (pH = 7.4), so that the amount of FBS in the mixture was 10%. The data represented as the mean ± SD (*n* = 3).

The *in vitro* stability was investigated by 10% FBS/PBS (pH = 7.4). After 48 h of co-incubation, the particle size of THD-NPs was changed to 85.21 ± 1.75 nm, and there was no significant change in the particle size at each time point compared to 0 h (Figure 5), which indicated that THD-NPs had good stability.

### Evaluation of pH-responsive release of THD-NPs *in vitro*

THD-NPs was set for sustained release and pH-responsive release. When the characteristics and stability of THD-NPs were revealed, we need to verify the release of DAS. This is one of the main parameters of the carrier.

**Figure 6.**
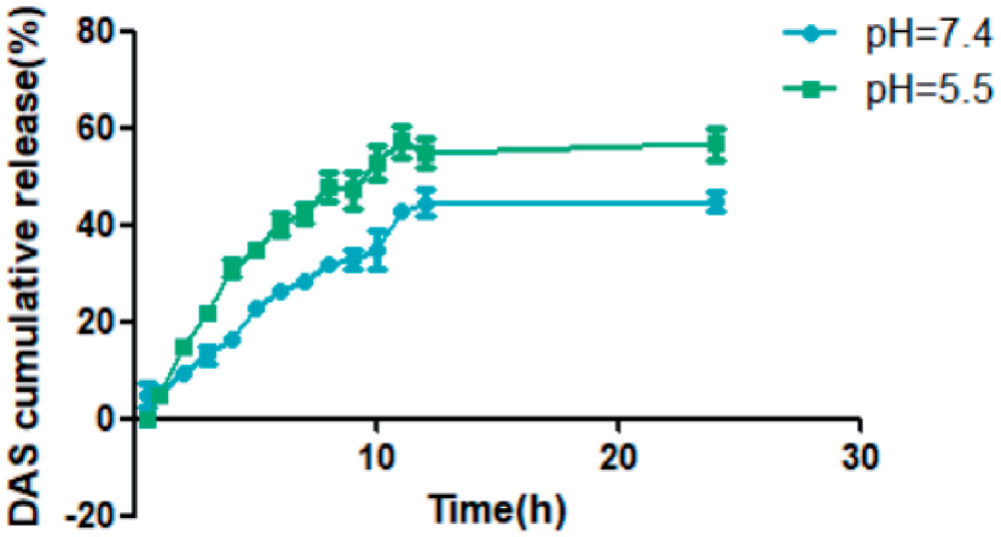
*In vitro* release of DAS at different pH values. The data represented the mean ± S.D. of three independent experiments.

In PBS buffer with pH = 7.4, THD-NPs showed slow drug release within 24 h, with a cumulative release rate of 44.93%. In PBS buffer with pH = 5.5, the release rate of THD-NPs was accelerated compared to pH = 7.4, and the cumulative release rate reached 56.75% (Figure 6).

As we all know, the pH of human blood is about 7.4, and the tumor tissues have a low pH due to abnormal metabolism of tumor cells. The above data indicated that THD-NPs has pH responsiveness, so that after intravenous administration, THD-NPs will not release all the drugs prematurely in the blood, but after reaching the tumor tissues, it will accelerate the release of the drugs, thereby realizing the targeted delivery and release of drugs.

### Uptake of tumor cells *in vitro*

**Figure 7.**
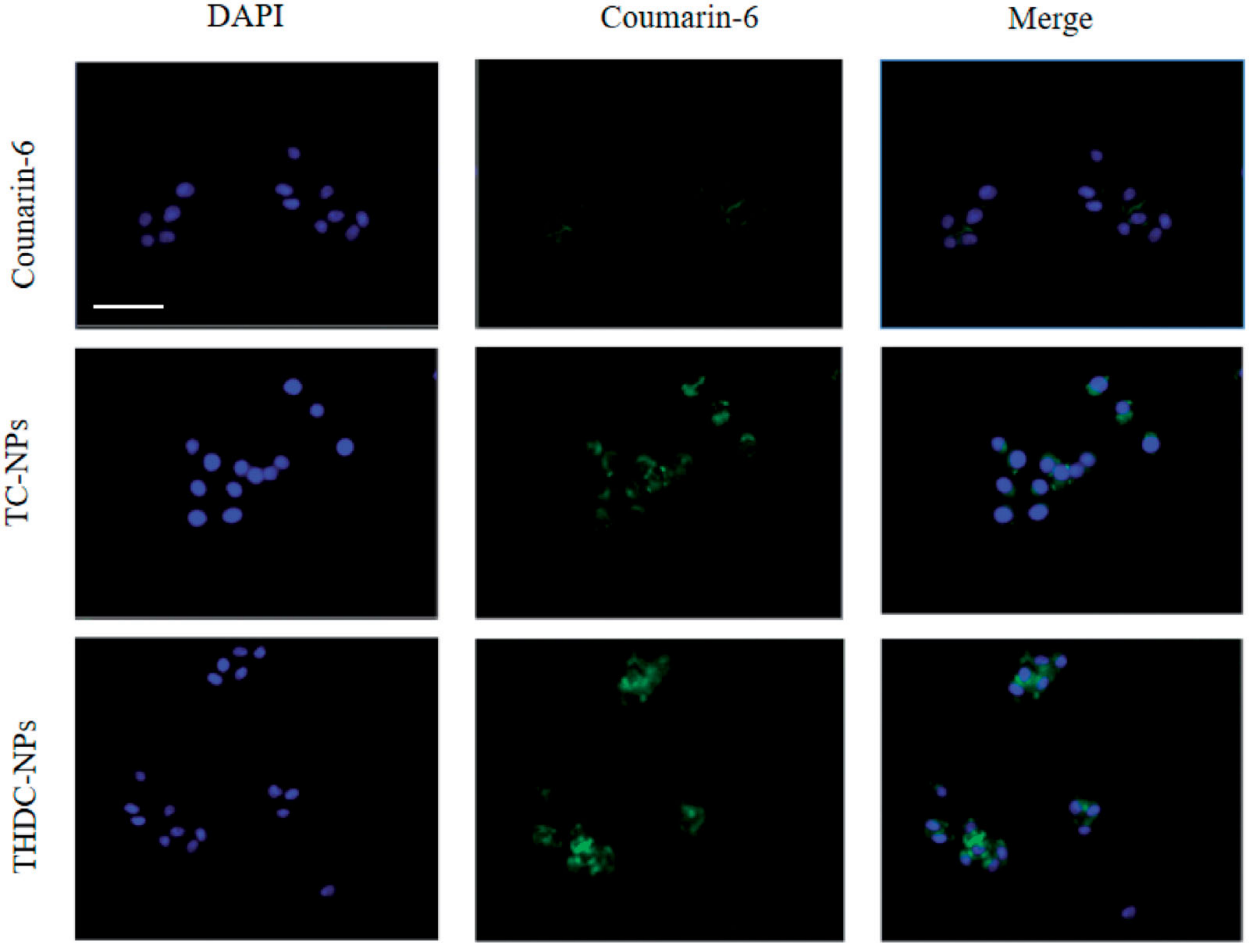
The images of HNE1/DDP cells after incubation with coumarin-6, TC-NPs, THDC- NPs for 2 h at 37 °C. Blue fluorescence represents live cells and green fluorescence represents Coumarin-6. Scale bar = 50 μm.

We need to investigate whether THD-NPs can exert its targeting effect on cancer cells, thereby increasing the intracellular accumulation of nanoparticles. Since THD-NPs does not have fluorescence, the fluorescent substance coumarin-6 was selected, TPGS, and TPGS/HA-DAS containing coumarin-6 (TC-NPs, THDC-NPs) were prepared according to the preparation method of THD-NPs. Our research showed that almost no fluorescence was seen in cells treated with free coumarin-6. Intracellular fluorescence increased slightly after TC-NPS treatment, which may be due to EPR effect. THD-NPs group showed the highest fluorescence, which proved that the addition of HA in the nanoparticle carrier could effectively promote THD-NPs nanoparticles to target cancer cells (Figure 7).

### Intracellular accumulation of drugs

**Figure 8.**
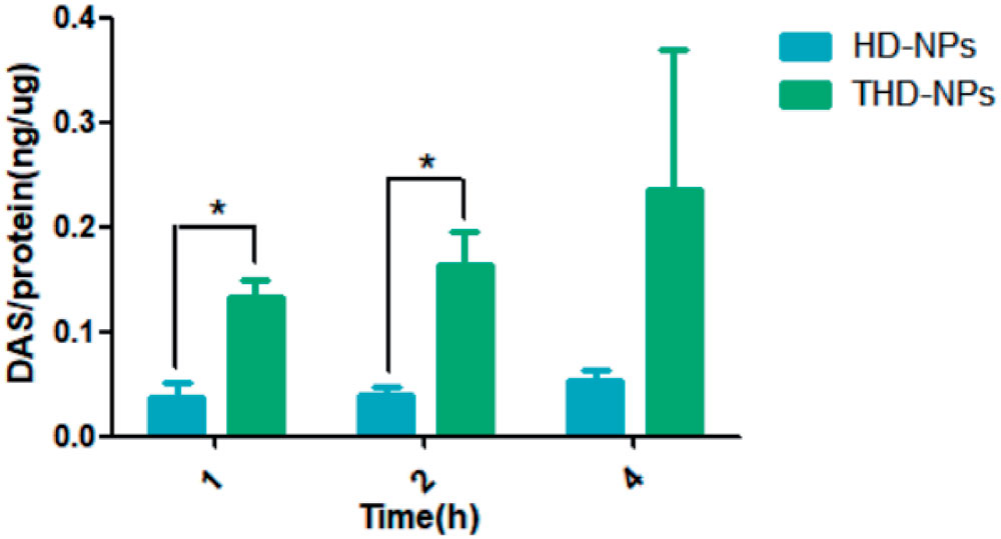
The accumulation of DAS in HNE1/DDP cells incubated with THD-NPs and HD-NPs for different times. The data represented as the mean ± SD (*n* = 3). *p < .05.

After confirming that nanoparticles can target tumor cells, we further explored the amount of intracellular drug accumulation caused by nanoparticles. The mechanism of HNE1/DDP cell resistance to cisplatin includes increased drug efflux caused by P-gp (Du et al., 2018). TPGS has been proven to inhibit P-gp activity, thereby improving cell MDR. We added TPGS to the carrier in order to improve the stability of the nanoparticles on the one hand, and on the other hand we hope that it can increase the accumulation of drugs in the cell (Huo et al., 2017). Therefore, we prepared HD-NPs (HA-DAS) without TPGS according to the preparation method of THD-NPs. Incubated both nanoparticles with HNE1/DDP cells simultaneously to detect the intracellular drug content at different time points. The results are shown in Figure 8.

### Cytotoxic effect of THD-NPs on both HNE1 and HNE1/DDP cell lines

**Figure 9.**
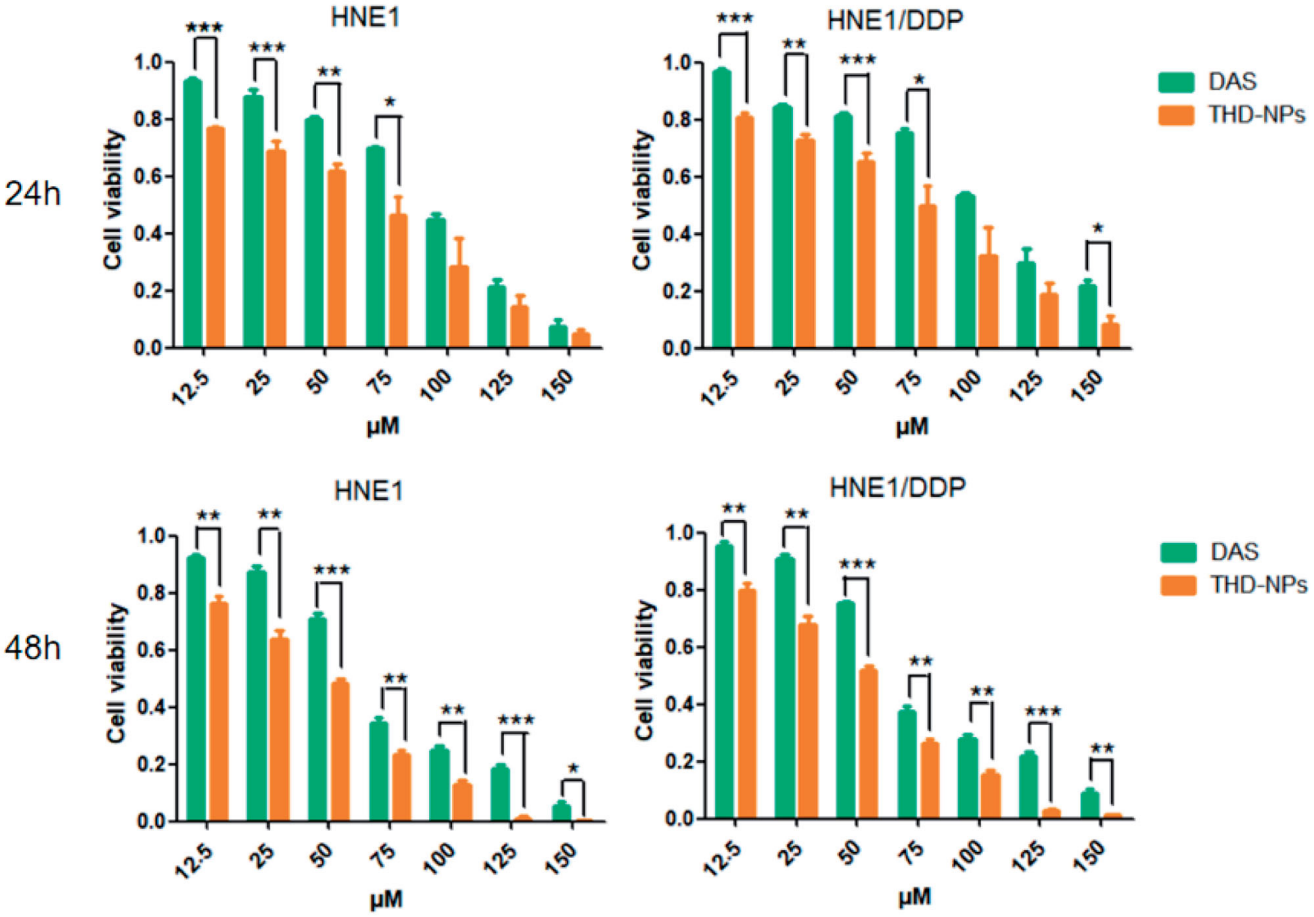
Cytotoxicity of different drug formulations in HNE1 and HNE1/DDP cells by MTT assays. Free DAS and THD-NPs on HNE1 (left panel) and HNE1/DDP (right panel) after 24 h and 48 h. The data represented as the mean ± SD (*n* = 3). ***p < .001, **p < .01, and *p < .05.

In order to detect the antitumor activity of THD-NPs *in vitro*, HNE1 cell lines and cisplatin-resistant HNE1/DDP cell lines were selected. In previous studies, HNE1/DDP cell lines have been proved to be typical cisplatin-resistant cell lines, and drug resistance mechanisms include drug efflux caused by P-gp. The above experiments have proved that THD-NPs can increase the amount of intracellular drugs by inhibiting the function of P-gp, and we further studied the proliferation inhibitory effect of THD-NPs on the two cell lines. After the free drug and THD-NPs acting on the two cell lines for different periods of time, the MTT results showed that after 48 h of action, the IC_50_ values of the free DAS on HNE1 and HNE1/DDP cell lines were 54.72 μM and 62.57 μM, respectively. After making it into nanoparticles, the IC_50_ values of HNE1 and HNE1/DDP cell lines were reduced to 30.99 and 35.03 μM, respectively. It is suggested that THD-NPs have more significant cytotoxic effects on the two cell lines (Figure 9; Table 2).

**Table 2.** IC_50_ of different drug formulations in HNE1 and HNE1/DDP cells.

Cell lines	IC_50_ (μM)			
	24 h		48 h	
	DAS	THD-NPs	DAS	THD-NPs
HNE1	70.18 ± 0.1040	45.24 ± 0.126**	54.72 ± 0.074	30.99 ± 0.010***
HNE1/DDP	101.21 ± 0.090	55.87 ± 0.174**	62.57 ± 0.082	35.03 ± 0.019***

The data represented as the mean ± SD (*n* = 3).

***p < .001, **p < .01 (DAS vs. THD-NPs).

### Investigation of anti-tumor effect of nanoparticles *in vitro*

**Figure 10.**
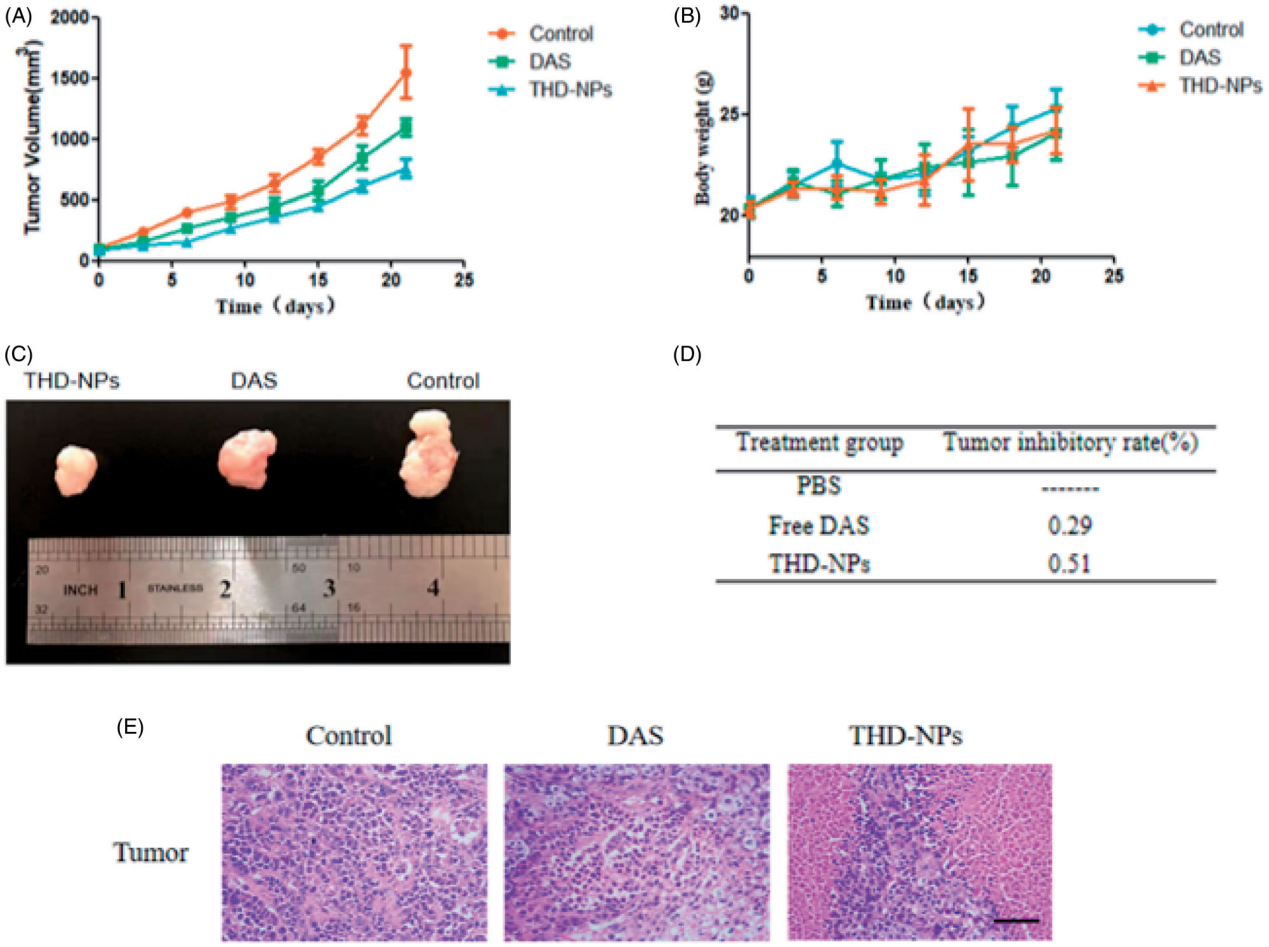
Evaluation of the anti-tumor effect of THD-NPs *in vivo*. (A) Tumor growth curve. Two weeks after inoculation with HNE1 cells, nude mice were injected intraperitoneally with equal amounts of saline, free DAS, and THD-NPs (5 mg/kg, calculated as DAS concentration), record the tumor weight and mouse body weight every 2 days during the administration period (B). (C) The tumor morphology of the mice in each group after the administration. (D) The tumor growth inhibition rate. The data are represented as the mean ± SD (*n* = 3). (E) H&E staining of the tumors after free DAS and THD-NPs treatment versus control. Scale bar = 50 μm.

Two weeks after the right upper limb of BALB/C nude mice was inoculated with HNE1 cells, the tumor volume reached about 100mm^3^, and the administration was started on the 12th day. Each nude mouse was injected with 200 μL of the different pharmaceutical preparation, and the dose was 5 mg/kg (calculated by DAS content). The results are shown in Figure 10.

**Figure 11.**
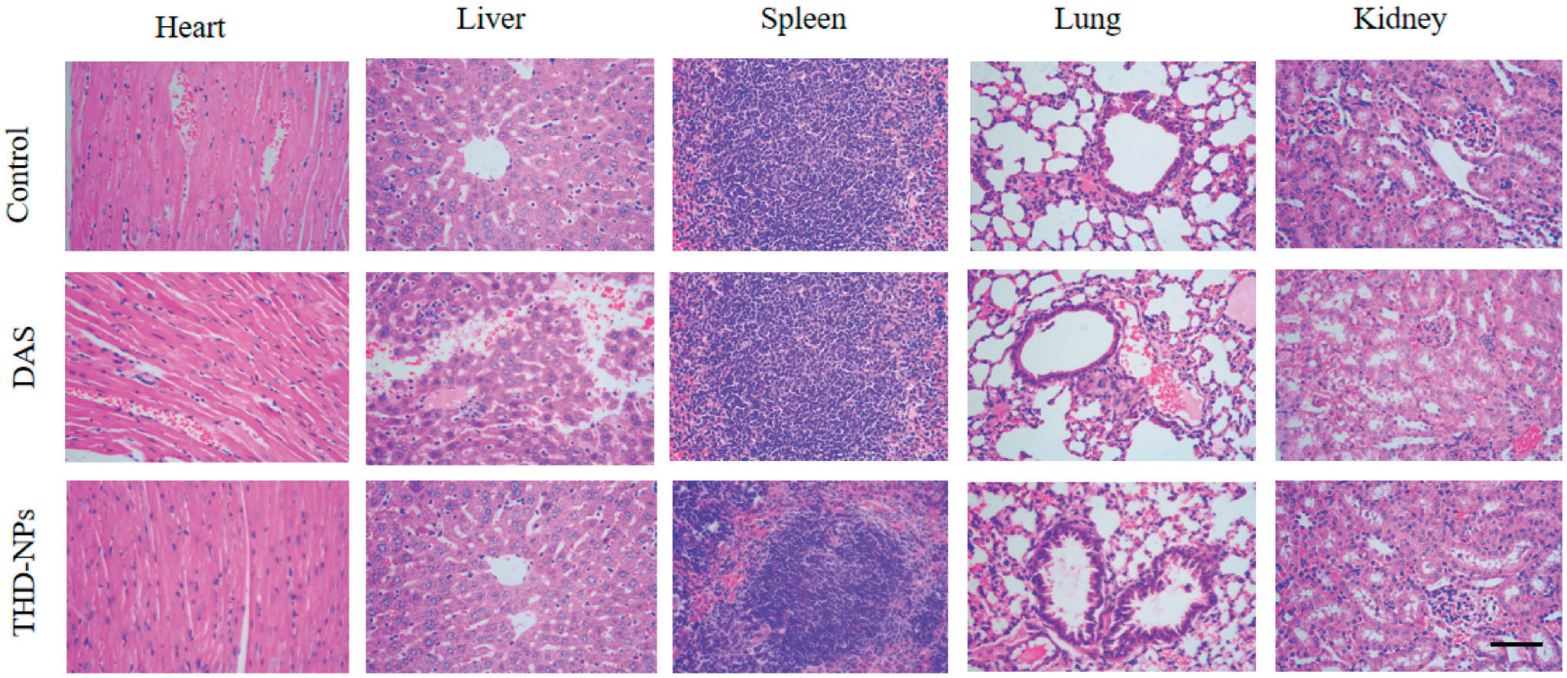
H&E staining of the heart, liver, spleen, lung, and kidney after free DAS and THD-NPs treatment versus control. Histological sections of heart, liver, spleen, lung, and kidney from the nude mice were stained with hematoxylin and counter-stained with eosin and microscopically analyzed for histopathological examinations of tissue toxicity. The images were taken at 400× objective lenses. Scale bar = 50 μm.

The weight changes of mice in each group were similar (Figure 10(B)), suggesting that the two treatments had no significant effect on the growth of mice. Tumor growth curve is shown in Figure 10(A), the average tumor volume of the saline group increased from 104.84 to 1550.63mm^3^, while the DAS group and THD-NPS group grew from 100.29 and 86.35mm^3^ to 1094.85 and 760.24 mm^3^, respectively. The results showed that after the mice were treated with free DAS and THD-NPS, compared with the saline group, both the DAS group and the THD-NPs group showed tumor suppression effect, and the antitumor effect of THD-NPs group was the most obvious (Figure 10(D)). The morphological appearance of the isolated tumor after the end of the administration also proved the above conclusion (Figure 10(C)). The results of HE staining of the tumor showed that compared with the control group, the cytoplasm of tumor cells in the DAS group disappeared, some of the nuclei were pyknotic, and inflammatory cells infiltrated. Large areas of necrosis and more apoptotic cells appeared in the THD-NPs group. It suggested that THD-NPs had significant tumor-suppressive effect. This result may be attributed to the active targeting effect produced by the specific binding of HA and CD44 protein and the passive targeting effect of nanoparticles through the EPR effect, thereby achieving targeted drug delivery and improving drug efficacy (Figure 11).

### Biosafety evaluation of THD-NPs

For a new drug preparation, safety is one of the most important factors. After it has been confirmed that THD-NPs have no significant effect on the body weight of mice during treatment, we need to evaluate the systemic toxicity caused by nanoparticles. Heart, liver, spleen, lung, and kidney tissues were sectioning, and histological morphology was observed by HE staining. Among them, the liver and kidney are the most important organs for drug metabolism. We focus on the liver and kidney toxicity caused by nanoparticles.

The pathological section results showed that, in the control group and THD-NPs group, the central hepatic vein was normal, and the hepatic cord was radial. In the DAS group, there was no obvious central vein, a large number of inflammatory cells were infiltrated, and some areas were necrotic. The glomeruli and tubules in the control group and THD-NPs group were normal, while the mesangial proliferation and sclerosis in the DAS group was accompanied by necrosis of tubule epithelial cells.

The above results showed that, compared with the control group, the DAS group had liver and kidney toxicity, and the THD-NPs group had a significant decrease in liver and kidney toxicity, which was similar to the control group. The results of other organs were the same as above. The above data all showed that the prepared THD-NPs had a protective effect on the hepatotoxicity of free drugs and had good biosafety.

## Conclusion

In this study, we have successfully constructed a safe and efficient drug delivery system that can achieve a slow and sustained pH-responsive release of drug, target tumor tissue, and potentially reverse MDR. The nanosystem takes DAS as part of the carrier, effectively improving the lipid solubility of the drug. Cell uptake experiments confirmed that the specific binding of HA and CD44 achieved specific targeting effect on tumor cells. The results of the *in vitro* release experiment showed that when the nanoparticles entered tumor cells through receptor-mediated endocytosis, the ester bond was broken, DAS-HA was degraded, and the drug was released. The intracellular drug accumulation experiments proved that the addition of P-gp inhibitor (TPGS) increased the intracellular drug concentration of drug-resistant cells and effectively reversed the MDR of tumor cells. *In vitro* cytotoxicity assays and *in vivo* antitumor experiments demonstrated that THD-NPs exhibited superior antitumor effects compared with free DAS. In conclusion, our study shows that HA-modified TPGS hybrid micelles can serve as effective carriers for DAS-targeted drug delivery and reversal of MDR.
